# MicroRNAs as biomarkers and molecular mediators of cognitive dysfunction in schizophrenia

**DOI:** 10.1007/s00702-025-02993-1

**Published:** 2025-08-08

**Authors:** Nabila M. Adly, Dalia Khalifa, Shaimaa Abdel-Ghany, Hussein Sabit

**Affiliations:** 1 Department of Medical Biotechnology, College of Biotechnology, Misr University for Science and Technology, P. O. Box 77, Giza, Egypt; 2 Psychiatry Department, Kasr Al Ainy Hospitals, Cairo University, Giza, Egypt; 3 Department of Environmental Biotechnology, College of Biotechnology, Misr University for Science and Technology, P. O. Box 77, Giza, Egypt

**Keywords:** Schizophrenia, Cognitive dysfunction, MiRNA, Biomarkers, Clinical diagnosis, Synaptic plasticity

## Abstract

Schizophrenia is a chronic psychiatric disorder characterized by positive, negative, and cognitive symptoms that impair daily functioning. Among these, cognitive dysfunction, affecting memory, attention, and executive function, is a core feature that lacks effective treatment. The clinical diagnosis of schizophrenia is contingent upon the Diagnostic and Statistical Manual of Mental Disorders, Fifth Edition (DSM-5), which is based on symptom assessment. However, DSM-5 criteria are subjective and lack biological specificity, leading to diagnostic delays and heterogeneity in patient classification. Emerging evidence implies that microRNAs (miRNAs), small non-coding RNAs that regulate gene expression post-transcriptionally, are integral to the molecular pathways contributing to cognitive dysfunction in schizophrenia. Dysregulated miRNAs impact neurodevelopment, synaptic plasticity, and neurotransmitter signaling, key processes implicated in cognitive impairment. Notably, miRNAs can be found in peripheral biofluids, making them promising non-invasive biomarkers for schizophrenia. Their potential diagnostic utility could enhance early detection and classification, overcoming the limitations of symptom-based clinical assessment. This review discusses the function of dysregulated miRNAs in schizophrenia-associated cognitive deficits, their molecular mechanisms, and their implications as biomarkers. Understanding miRNA-mediated regulation of cognitive function could open the door for innovative diagnostic tools and personalized interventions, ultimately improving patient outcomes.

## Introduction

Schizophrenia (SCZ) is a complex and impactful neuropsychiatric disorder affecting approximately 1% of people worldwide (Mosquera et al. 2024). Its trajectory is complex and varied due to its wide range of mental symptoms, which include complex expressions of cognitive, emotional, and behavioral abnormalities (Li et al. 2024). Because schizophrenia is inherently heterogeneous, there is disagreement over its pathophysiology, etiology, and diagnostic standards (Tandon et al. 2024).

The evolution of diagnostic criteria has undergone significant revisions since Emil Kraepelin’s early 20th-century descriptions of dementia praecox. Initially categorized by subtypes such as paranoid, disorganized, and catatonic, the DSM-5 eliminated these distinctions in 2013 due to their limited clinical utility and reliability. Instead, the current framework adopts a spectrum-based approach, acknowledging the heterogeneity of symptom presentation and severity across individuals. This shift reflects growing evidence that symptom clusters in schizophrenia overlap with other psychotic disorders, such as schizoaffective disorder and delusional disorder, necessitating dimensional assessments for accurate diagnosis (Shmukler Шмуклер 2021).

The diagnosis of schizophrenia currently depends heavily on clinical observation of symptoms and behaviors. This subjective approach can lead to misdiagnosis, delayed treatment, and inconsistent outcomes. Therefore, searching for objective, reliable biomarkers is crucial for improving diagnostic accuracy and facilitating earlier interventions. MicroRNAs have emerged as promising candidates for such biors in various diseases, including schizophrenia (Li et al. 2024).

Given their stability in biofluids and their regulatory capacity, miRNAs have shown promise as biomarkers for schizophrenia. Their utilization could enhance diagnostic precision, facilitate early intervention, and lead to more personalized therapeutic strategies. This review provides an integrated and up-to-date synthesis of the mechanistic pathways linking miRNAs to cognitive dysfunction in schizophrenia, with particular emphasis on their potential as biomarkers and therapeutic targets. It organizes the evidence by focusing on specific miRNAs implicated in key neurobiological processes, offering a comprehensive overview of how dysregulated miRNAs contribute to neurodevelopmental, synaptic, and neurotransmitter abnormalities associated with the disorder. Furthermore, the review discusses the current challenges and future directions for translating miRNA research into clinical practice, distinguishing it from previous literature in the field.

## Cognitive dysfunction in schizophrenia

A complex mental illness, schizophrenia is typified by a wide range of symptoms that cut across several different areas. Positive symptoms like delusions and hallucinations, negative symptoms like anhedonia, alogia, avolition, and social disengagement, and cognitive impairments that impact working memory, verbal and visuospatial learning, attention, processing speed, problem-solving, and cognitive flexibility comprise the core symptomatology (Moura et al. 2022; McCutcheon et al. 2020).

**Fig. 1:**
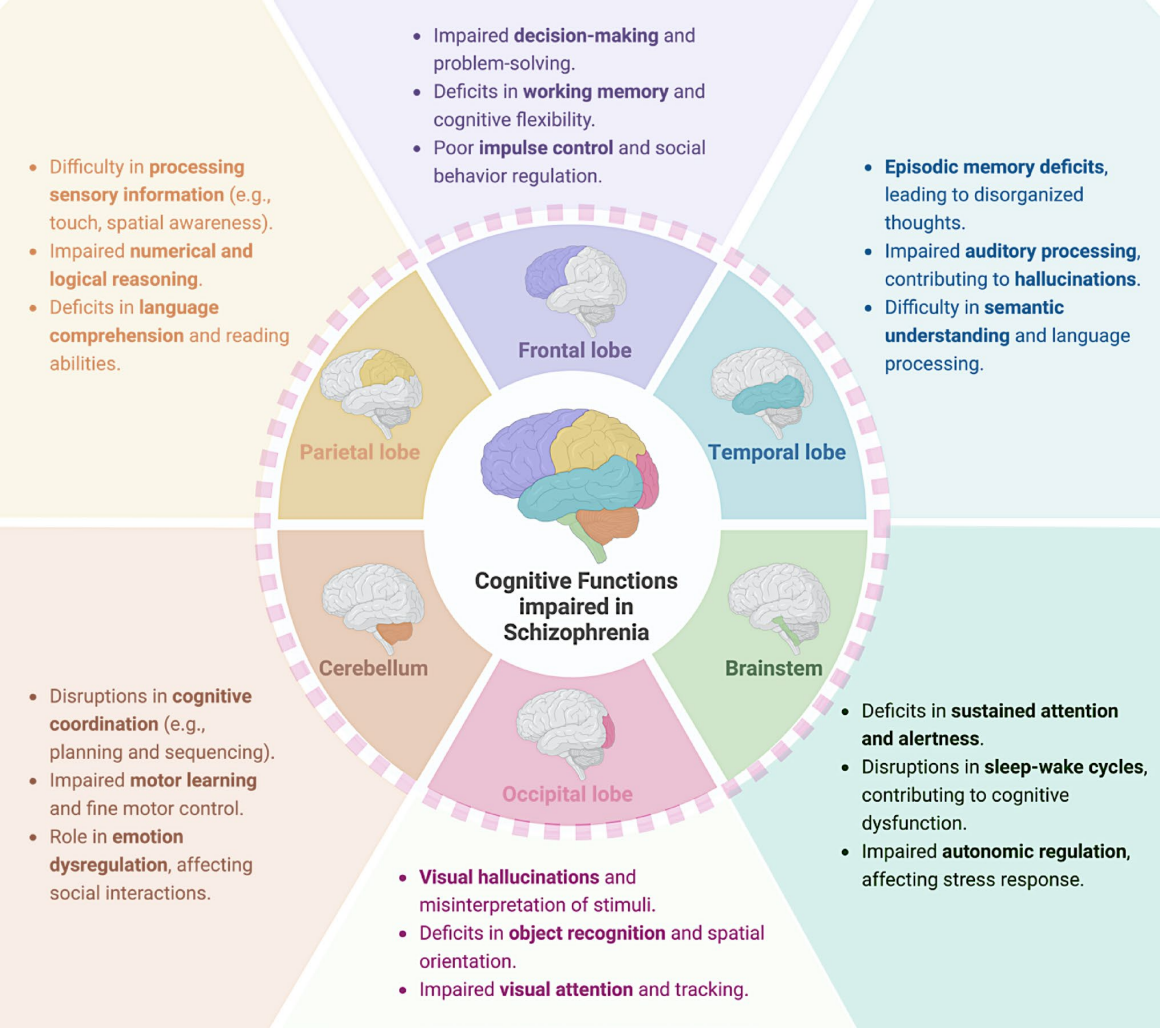
Cognitive Deficits in Schizophrenia: Affected Brain Regions and Functions. This illustration highlights key brain regions involved in cognitive dysfunction in schizophrenia. The frontal lobe is responsible for executive functions, decision-making, and behavioral control, with impairments leading to poor problem-solving, working memory deficits, and impulse dysregulation. The parietal lobe contributes to sensory perception, language comprehension, and mathematical reasoning, and its dysfunction can result in difficulties in spatial awareness and logical processing. The temporal lobe plays a crucial role in memory formation and language processing, with deficits contributing to disorganized thoughts and auditory hallucinations. The occipital lobe, essential for visual perception and spatial orientation, is often affected, leading to visual misinterpretations and impaired object recognition. Furthermore, the cerebellum—which has historically been linked to motor coordination—is becoming more widely acknowledged for its function in regulating emotions and cognition, with dysfunction leading to cognitive disarray

Additionally, the functional outcomes of people with schizophrenia are significantly impacted by deficiencies in social cognition, which include emotional intelligence, facial emotion recognition, emotion evaluation, and social inference. These impairments adversely affect interpersonal relationships, hinder community integration, and limit vocational success, thereby contributing to long-term disability and reduced quality of life (Orsolini et al. 2022) (Fig. 1).

A key component of schizophrenia is cognitive impairment, which impacts a variety of areas, including working memory, attention, processing speed, verbal learning and memory, and executive functions. These deficits often precede the onset of overt psychotic symptoms and remain relatively stable throughout the illness, significantly impacting patients’ functional outcomes and quality of life (McCutcheon et al. 2023; Seitz-Holland et al. 2022). The brainstem, which regulates arousal and attention, may also be affected, leading to disturbances in sustained focus and sleep-wake cycles. Together, dysfunction across these brain regions contributes to the widespread cognitive impairment found in schizophrenia.

The assessment of cognitive deficits in schizophrenia typically involves standardized neuropsychological tests. The popular MATRICS Consensus Cognitive Battery (MCCB) assesses seven cognitive domains: verbal learning, visual learning, working memory, reasoning and problem-solving, social cognition, attention/vigilance, and processing speed. While the Wechsler Adult Intelligence Scale-Third Edition (WAIS-III) measures general cognitive ability, other instruments, such as the Wisconsin Card Sorting Test (WCST) and the Trail Making Test-Part B (TMT-B), evaluate executive functions. Furthermore, the Positive and Negative Syndrome Scale (PANSS) does not directly evaluate cognitive function; instead, it assesses the severity of symptoms (Nuechterlein et al. 2024).

Current diagnostic frameworks, such as the DSM-5 and the ICD-11, still rely largely on subjective clinical assessment of symptoms and functional impairment. While these criteria are essential, they lack biological specificity and may not capture early cognitive changes or subtle pathophysiological processes (Park 2024; Gaebel et al. 2020).

Consequently, there is a pressing need for objective biomarkers to enhance diagnostic accuracy and treatment monitoring. Biomarkers could facilitate early detection, predict treatment response, and enable personalized therapeutic interventions, thereby improving patient outcomes (Martinez and Peplow 2024).

## Role of miRNAs in a healthy brain

MicroRNAs (miRNAs) are a class of small non-coding RNAs that are essential for both development and disease. They are transcribed from endogenous genomic regions—sometimes located in intergenic regions, exons, or introns—into long primary transcripts (pri-miRNAs), which are then processed into mature miRNAs of approximately 18–25 nucleotides in length (Mohamed and Freude 2024).

### miRNAs biogenesis

The nucleus is where miRNA biosynthesis starts, and the cytoplasm is where mature miRNAs are made. Primary miRNAs (pri-miRNAs), which have one or more hairpin structures and can be up to thousands of nucleotides long, are created by capping, splicing, and polyadenylation after miRNAs are primarily transcribed via RNA polymerase II-dependent transcription (Tielke et al. 2022). Pri-miRNAs are converted into precursor hairpins by the Drosha–DiGeorge Syndrome Critical Region 8 (DGCR8) complex, about 70 nucleotides long. Exportin-5 then translocates these precursor hairpins into the cytoplasm through the nuclear pore (Khavari and Cairns 2020).

Pre-miRNAs in the cytoplasm are broken down into double-stranded RNA duplexes by an RNA enzyme known as Dicer, or miRNA strands and their complementary sequences (miRNA/miRNA*). MiRNAs orchestrate post-transcriptional gene silencing by targeting the 3′ untranslated region (UTR), focusing on the seed region at the 5′ end that spans nucleotides 2–7 (Muñoz-Velasco et al. 2024). While the other miRNA* strand is degraded, mature miRNAs can attach to the Ago-2-containing RNA-induced silencing complex (RISC) and mediate gene silencing, causing mRNA breakage or translational repression (Suster and Feng 2021).

miRNAs are a key regulator of fundamental biological processes that control apoptosis, proliferation, and cell differentiation. MiRNAs indirectly control pathophysiological states and suppress downstream gene expression, primarily through transcriptional inhibition and mRNA cleavage or degradation (Li et al. 2024). Through various pathways, miRNAs control mRNA expression by binding to complementary target sequences in mRNA, interfering with translation processes, and changing or stopping the synthesis of protein products. When miRNAs bind to their target mRNAs, mRNA attenuators also bind, which causes mRNA instability, reduced expression, and even degradation (Narayanan and Schratt 2020).

MiRNAs have attracted much research attention because of their critical role in brain function and neuronal development. The active role of miRNAs might be implicated in the pathophysiological mechanisms of schizophrenia (Zhang et al. 2023; Amoah et al. 2020).

**Fig. 2:**
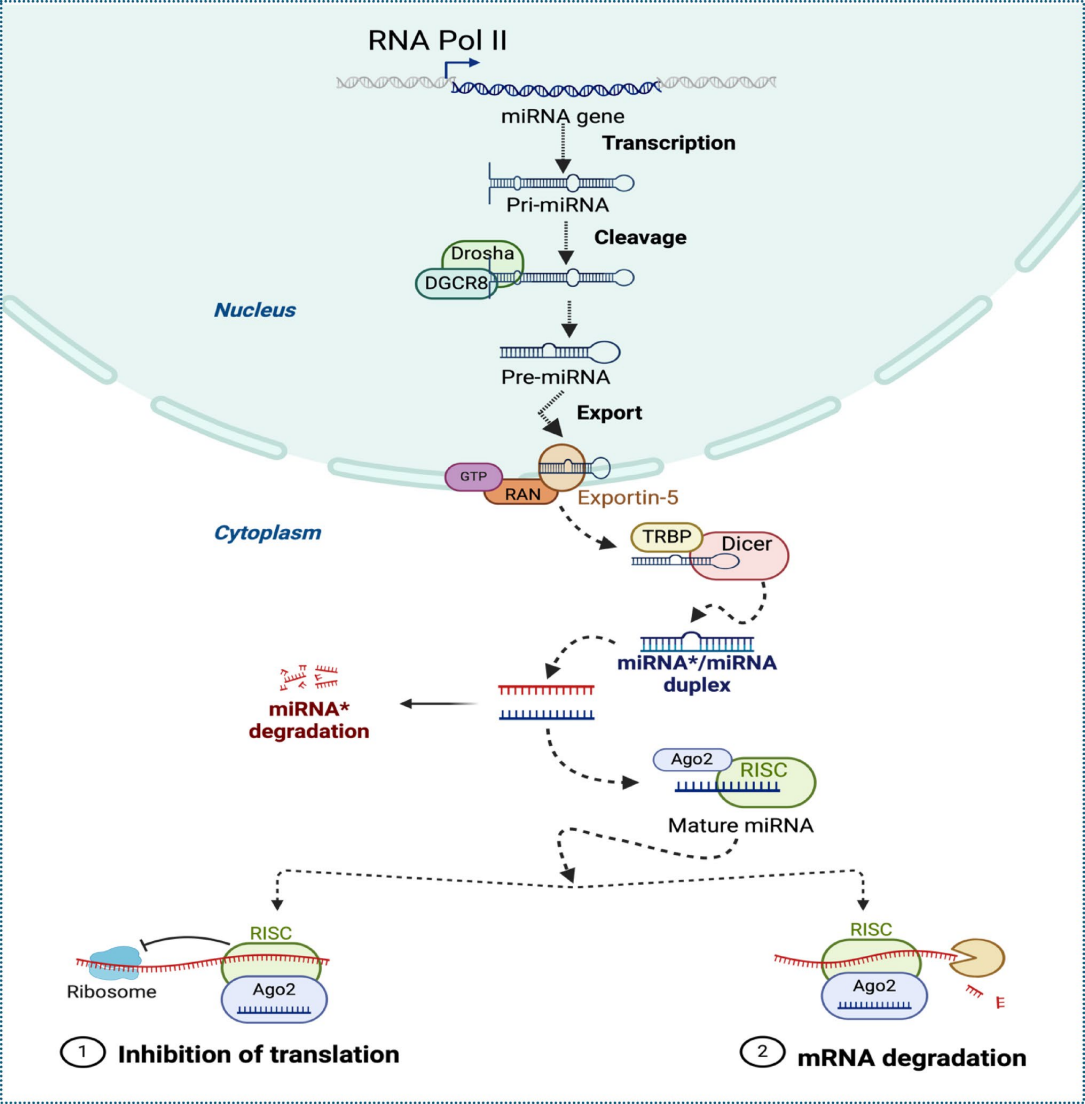
Diagrammatic representation of the biogenesis and function of miRNA. RNA polymerase II/III transcription, which is transcribed from genomic DNA into primary transcripts (pri-miRNAs), is typically the first step in the biogenetic pathway and associated roles of miRNAs in cells. In the nucleus, Dorsha/DGCR8 cleaves pri-miRNA to create miRNA precursors (pre-miRNA), which are then transported to the cytoplasm by exportin-5 and further converted by Dicer/TRBP into double-stranded RNA: miRNA*/miRNA. While the complementary strand, miRNA*, degrades, mature miRNAs can bind to RNA-induced silencing complex (RISC) assemblies and direct the translational repression of target mRNA

New non-canonical biogenesis pathways that only involve Dicer and not the microprocessor complex have been found in addition to this canonical pathway. Nevertheless, it is still unknown how many miRNAs this pathway generates (Narayanan and Schratt 2020). miRNA biogenesis process is depicted in Fig. 2.

Despite being present throughout the human genome, 70% of miRNAs are expressed in the brain, suggesting that they are crucial for brain development. miRNAs are regulators of genes. Numerous miRNAs can control the expression of a single gene, but a single miRNA can target the expression of many different genes (Zhang et al. 2023).

As miRNAs precisely regulate gene expression at different stages of neurodevelopment, they influence numerous aspects of neuronal development, including the proliferation of neural progenitor cells, neuronal fate determination, circuit formation, synaptic plasticity, and preserving the overall brain health (Wang et al. 2022a, b).

For a healthy brain to develop properly, a healthy miRNA biogenesis process must be maintained. Brain disorders, including SCZ, have been linked to any interference with the biogenesis of miRNAs (Hu et al. 2019).

## miRNA-mediated regulation of dopaminergic pathways in schizophrenia

miRNAs play a crucial role in neurotransmission and neural signaling by modulating pathways such as dopaminergic, glutamatergic, and GABAergic signaling, which collectively contribute to the clinical manifestations of schizophrenia (Li et al. 2024). While the dopamine hypothesis has historically provided a foundation, proposing that mesolimbic hyperdopaminergia underlies positive symptoms and mesocortical hypodopaminergia contributes to cognitive and negative symptoms, current evidence highlights a more complex interplay involving glutamate dysregulation, GABAergic inhibitory deficits, neuroinflammation, and synaptic plasticity abnormalities (McCutcheon et al. 2020, 2023). MicroRNAs (miRNAs) play significant roles in the molecular mechanisms underlying schizophrenia. Table 1 summarizes specific miRNAs, their associated cellular/molecular events, related pathological conditions, and corresponding citations.

Among the key regulators of dopaminergic signaling, microRNA-9-5p (miR-9-5p) has garnered significant attention due to its direct targeting of the dopamine D2 receptor (DRD2). DRD2 encodes for the type-2 dopamine receptor (D2R), an essential G-coupled presynaptic receptor of the dopaminergic system. Overexpression of miR-9-5p reduces striatal D2 receptor density, highlighting its critical role in modulating dopaminergic neurotransmission. Given that dysregulations in dopamine signaling are fundamental to schizophrenia, the ability of miR-9-5p to modulate DRD2 expression is particularly significant.

**Table 1:** MiRNAs linked to brain cellular and molecular processes, their target genes, and their functions in pathological conditions, especially schizophrenia, are listed along with relevant citations

miRNA	Target genes	Molecular & cellular roles	Pathological role in Schizophrenia	Citations
miR-137	TCF4, CACNA1C, CRTC1	Neural differentiation, synaptic plasticity, neuronal migration	Dysregulated neurodevelopment, cognitive deficits, and risk factors for schizophrenia	(Thomas and Zakharenko 2021; Wright et al. (2013)
miR-9-5p	TLX, FOXG1, REST, NFkβ, SLC20A2, FBN2	Neurogenesis, Neural differentiation, Neuroinflammation, Transcription activity	Enrichment in schizophrenia risk genes, suggesting a role in disease etiology.	(Ilieva 2024; Paiva et al. 2017; Hauberg et al. 2016; Yao et al. 2014; Coolen et al. 2013)
miR-181b	BCL2, SIRT1	Apoptosis regulation, neuronal differentiation	Patients with schizophrenia may have altered expression in their temporal cortex, indicating a pathophysiological role	(Li et al. 2024; Ghafouri-Fard et al. 2021; Beveridge et al. 2008)
miR-134	LIMK1, CREB, Pumilio	Dendritic spine development, synaptic plasticity	Upregulated in schizophrenia; implicated in synaptic dysfunction	(Baby et al. 2020; Leontariti et al. 2020; Zampa et al. 2018; Beveridge et al. 2010; Schratt et al. 2006)
miR-144	IRS1	Insulin signaling, erythropoiesis	Recognized as a possible target for the therapeutic intervention of schizophrenia	(Rizos et al. 2016; Wang et al. 2014)
miR-132	CREB, MeCP2, AchE, FOXO3	Synaptic plasticity, neuronal morphogenesis, Inflammation, and Oxidative stress	Dysregulation is linked to impaired synaptic function, neurodegeneration, and cognitive deficits	(Fatimy et al. 2018; Mishra et al. 2017; Yu et al. 2015; Wong et al. 2013; Miller et al. 2012)
miR-432	CHAC1, E2F3, P55PIK, KEAP1, NESTIN	Oxidative stress response, cell cycle regulation, myogenesis, neuronal differentiation, and development	Involvement in vital cellular functions like oxidative stress response and cell cycle regulation, which may affect neuronal viability and function, and lead to negative symptoms and neurocognitive deficits in schizophrenia	(Honorato-Mauer et al. 2023; Akdemir et al. 2017; Ma et al. 2017; Chen et al. 2016)
miR-130b	ERK, AKT, PTEN, MECP2	Neuronal development, synaptic plasticity, neuroinflammation, dopamine signaling	Dysregulated PI3K/AKT pathway, altered neurotransmission, increased vulnerability to schizophrenia	(Lei et al. 2021; Li et al. 2017; Wei et al. 2015)
miR-34a	CACNB3, COMT, BCL2, SIRT1	Regulation of apoptosis, neuronal differentiation, and synaptic plasticity	Dysregulation is linked to impaired neuronal development and increased apoptosis and neurodegeneration; potential biomarker for schizophrenia	(Lai et al. 2016; Bavamian et al. 2015)
miR-195	BACE1, APP, BDNF, RELN, VSNL1	dendritic growth, spine formation, synaptic plasticity, neuronal migration	Dysregulation linked to neuropsychiatric disorders and cognitive deficits observed in schizophrenia	(Li et al. 2024; Beveridge et al. 2010; Pan et al. 2021; Mellios and Sur 2012)

Beyond its role in dopaminergic regulation, miR-9-5p is essential for neurodevelopment, aligning with the established biological mechanisms underlying schizophrenia. It serves as a pivotal regulator of neuronal differentiation and interacts with Fragile X Mental Retardation Protein 1 (FXR1), forming a regulatory feedback loop that influences schizophrenia pathology (Mohamed and Freude 2024; Hauberg et al. 2016).

The primary coding gene of miR-9-5p, MIR9-2, is situated within a highly pleiotropic genome-wide risk gene linked to significant psychiatric characteristics at the genetic level (Liu et al. 2020a, b). Additionally, schizophrenia susceptibility has been linked to the MIR9-2 host gene, LINC00461 (Rao et al. 2022). Furthermore, biological processes like the development of the nervous system, the regulation of neuronal differentiation, and the neurotrophin signaling pathway are all impacted by miR-9-5p target genes (Jin et al. 2022).

Further insights into miR-9-5p biogenesis highlight its interaction with DiGeorge Syndrome Critical Region 8 (DGCR8). According to Nogami, Miyamoto (Nogami et al. 2021), DGCR8 in mammals can efficiently process pri-miR-9-2 to stimulate the production of miR-9. It is thought that the DGCR8-miR-9-2 axis plays a role in the development of schizophrenia and neurogenic differentiation. Furthermore, DiGeorge syndrome, a known risk factor for schizophrenia, is caused by the deletion of the DGCR8 gene on chromosome 22q11, and patients often have cognitive and behavioral impairments.

From a clinical perspective, Fu, Baranova (Fu et al. 2023) reported that miR-9-5p expression levels were significantly downregulated in schizophrenia patients’ peripheral blood as opposed to that of healthy controls. Additionally, 24 of the 1136 genome-wide schizophrenia risk genes were regulated by miR-9-5p, significantly higher than expected by chance. Literature-based analysis suggests that miR-9 exerts predominantly inhibitory effects on schizophrenia-related genes (e.g., IL1B, ABCB1, FGFR1) while promoting the expression of INS, indicating a potential protective role of miR-9 against schizophrenia.

Additionally, patients with first-episode schizophrenia have been found to have altered expression levels of miR-9-5p, indicating that the protein may serve as a biomarker for the illness. This is consistent with research showing miR-9-5p is enriched among miRNAs that control genes linked to schizophrenia risk, highlighting its significance in the molecular landscape of the illness (Jin et al. 2021).

Research has shown that miR-9-5p is not only involved in the regulation of DRD2 but also interacts with other genes associated with schizophrenia risk. For instance, it has been linked to the modulation of glycogen synthase kinase 3B (GSK3B) signaling, which is crucial for neuronal function with significant implications for neurodevelopmental processes and neuropsychiatric disorders and has been implicated in the etiology of schizophrenia (Camkurt et al. 2016).

miR-9-5p has been shown to directly target GSK3B mRNA, decreasing this kinase’s expression. This interaction is pivotal in modulating neuronal morphology and function. For instance, Liu, Zuo (Liu et al. 2020a, b) demonstrated that miR-9-5p inhibits mitochondrial damage and oxidative stress in Alzheimer’s disease cell models by targeting GSK3B, promoting neuronal survival.

The miR-9-5p/GSK3B axis plays a crucial role in neurodevelopment. Mingardi, La Via (Mingardi et al. 2021) found that miR-9-5p directly influences neuronal morphology, with its upregulation rescuing stress-induced dendritic shortening in hippocampal pyramidal neurons. This suggests that miR-9-5p-mediated regulation of GSK3B is essential for maintaining dendritic architecture under stress conditions.

Additionally, the dysregulation of miR-9-5p has been associated with inflammatory processes, which may further complicate the neurobiological underpinnings of schizophrenia. Studies have indicated that miR-9-5p can negatively regulate the NF-κB signaling pathway, a critical mediator of inflammation, thereby linking inflammatory responses to the dysregulation of dopaminergic signaling in schizophrenia (Zhao et al. 2020).

This suggests that miR-9-5p may play a dual role in both neuronal differentiation and inflammatory regulation, potentially influencing the severity and manifestation of schizophrenia symptoms. In conclusion, while miR-9-5p is a promising candidate for understanding the biological underpinnings of schizophrenia, it requires additional validation before being considered a robust diagnostic biomarker.

Single nucleotide polymorphisms (SNPs) could interfere with miRNA-mRNA interactions, which can change the way schizophrenia risk genes are regulated and affect a person’s vulnerability to the disorder. The single nucleotide polymorphism (SNP) rs1130354 is located within the 3’ untranslated region (3’ UTR) of the dopamine receptor D2 gene (DRD2) (Mohamed and Freude 2024). This SNP can disrupt the binding of microRNA-326 (miR-326) to the DRD2 mRNA, leading to altered gene expression. Specifically, the rs1130354 variant interferes with miR-326-mediated repression of DRD2, potentially resulting in increased DRD2 expression (Carneiro et al. 2024). This dysregulation has been linked to an increased risk of schizophrenia development.

Emerging research highlights the direct interaction between miR-34a and the catechol-O-methyltransferase (COMT) gene, wherein miR-34a downregulates COMT expression. Studies further support this regulatory mechanism by demonstrating that both miR-34a and miR-30a-5p suppress COMT gene expression in cellular models of schizophrenia (Tonk et al. 2024).

The COMT enzyme plays a critical role in dopamine metabolism, particularly in the prefrontal cortex, where it facilitates dopamine degradation. Upregulation of miR-34a leads to COMT inhibition, thereby altering dopamine catabolism. Reduced COMT activity results in elevated dopamine levels, which may disrupt normal neurotransmission and contribute to schizophrenia pathophysiology, potentially exacerbating symptoms associated with the disorder. Given that dopamine dysregulation is a well-established hallmark of schizophrenia, miR-34a-mediated alterations in COMT expression could have profound implications for disease progression and symptom severity (Tonk et al. 2024).

Due to its altered expression patterns in schizophrenia, miR-34a has been proposed as a potential biomarker for the disorder. Notably, its aberrant expression has been observed in PBMCs of individuals with schizophrenia. However, differences in miR-34a expression between the brain and peripheral circulation complicate its use as a direct indicator of central nervous system activity. Further research is required to determine the diagnostic utility of miR-34a and its role in schizophrenia pathophysiology (Lai et al. 2016; Rodrigues et al. 2024).

While miR-30a-5p and miR-34a-5p have been shown to decrease COMT expression, another miRNA, miR-148b-3p, is noted for its role in regulating COMT indirectly by targeting the ZNF804A gene, which in turn affects COMT expression in human neuroblastoma cells (Wu et al. 2020).

## Role of miR-137 in synaptic plasticity and neural development

**Fig. 3:**
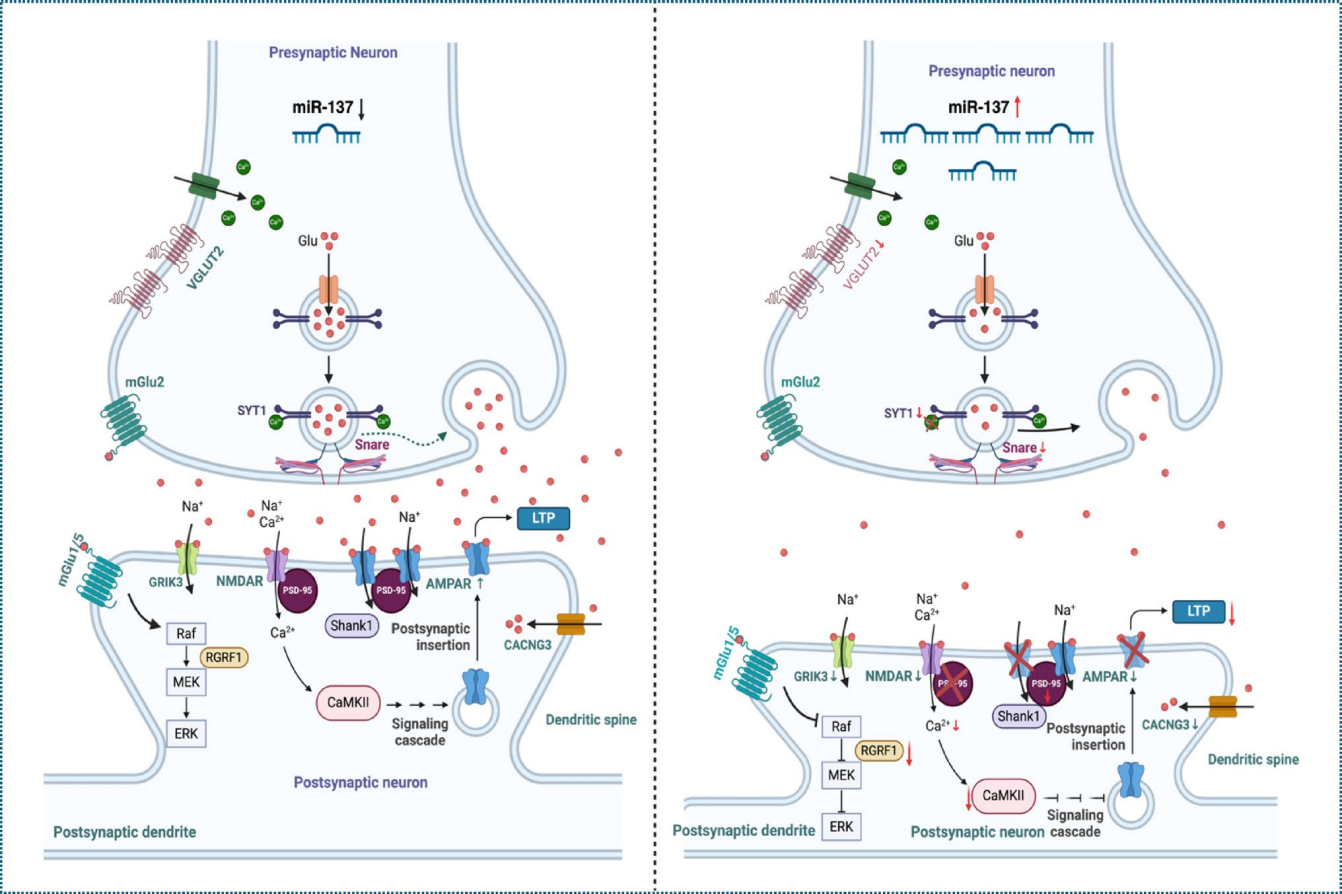
miR-137-Mediated Synaptic Dysfunction in Schizophrenia: Impaired Glutamatergic Transmission and Structural Alterations. (Left) In the normal synapse, presynaptic glutamate is properly packaged by vesicular glutamate transporter 2 (VGLUT2) and released into the synaptic cleft. Postsynaptically, AMPA receptors (AMPARs) and NMDA receptors (NMDARs) are well-expressed and stabilized by scaffolding proteins such as SHANK, PSD-95, and CACNG3. Efficient NMDAR signaling leads to calcium influx, Ras-ERK activation, and long-term potentiation (LTP), supporting synaptic plasticity and cognitive function. (Right) In schizophrenia, synaptic dysfunction is characterized by disrupted glutamate transmission and reduced expression of AMPARs, NMDARs, and CACNG3, leading to impaired synaptic strength. Loss of SHANK and PSD-95 weakens receptor anchoring, contributing to dendritic spine shrinkage and synaptic instability. Impaired NMDAR-mediated calcium influx and weakened Ras-ERK signaling result in deficient synaptic plasticity, a key feature of schizophrenia pathology. Downward arrows (↓) indicate reduced expression or function of the respective proteins.

miR-137 is a microRNA located within the schizophrenia-associated genomic region MIR137HG. It targets genes and is essential for controlling synaptic plasticity, such as NRGN and CACNA1C, which are involved in neural development and synaptic function, contributing to the neurodevelopmental hypothesis of schizophrenia. Genome-wide association studies (GWAS) have implicated miR-137 in schizophrenia, showing that variations in its expression can affect synaptic function, leading to cognitive deficits and psychotic symptoms associated with the disorder (Pergola et al. 2024; He et al. 2018) (Fig. 3).

miR-137 influences synaptic plasticity through multiple mechanisms. Overexpression of miR-137 modifies short-term plasticity and decreases basal synaptic transmission. It impairs long-term potentiation (LTP), a critical process for learning and memory, by affecting presynaptic function (Hu and Li 2017). Furthermore, miR-137 overexpression reduces synapse formation by approximately 31%, impacting dendrite growth and synapse density in a development-dependent manner. This results in ultrastructural changes, including reduced active zone length, decreased total vesicle number, and altered distribution of synaptic vesicles, which collectively contribute to impaired synaptic transmission (He et al. 2018; Howell and Law 2020). Additionally, miR-137 mediates metabotropic glutamate receptor (mGluR)-dependent long-term depression (LTD), another form of synaptic plasticity (Kaurani 2024). Inhibition of mossy fiber LTP in the hippocampus further underscores miR-137’s role in synaptic modulation.

miR-137 regulates key schizophrenia-associated genes, including NRGN and CACNA1C, affecting neural development and function (Liu et al. 2024). The RISC binds to the 3′ untranslated region (3′ UTR) of target mRNAs to cause degradation or prevent translation. NRGN (Neurogranin) is a postsynaptic protein crucial for synaptic plasticity and learning, regulating synaptic strength and cognitive processes. miR-137’s regulation of NRGN may impact synaptic function and contribute to neurodevelopmental disorders like schizophrenia (Wright et al. 2013). Similarly, CACNA1C encodes an L-type calcium channel subunit essential for neuronal excitability and synaptic transmission. Variations in CACNA1C are linked to psychiatric disorders, including schizophrenia, and miR-137’s interaction with CACNA1C may modulate calcium influx into neurons, influencing synaptic plasticity and neuronal excitability (Mokhtari et al. 2022; Guan et al. 2014).

By regulating NRGN and CACNA1C, miR-137 influences synaptic plasticity, essential for memory and learning. Dysregulation of these interactions can impair synaptic function and contribute to the pathophysiology of schizophrenia (Pacheco et al. 2019). Additionally, miR-137 is involved in glutamatergic synaptic transmission, regulating presynaptic targets associated with neurotransmitter release and several glutamatergic receptors, such as GluA1 and GluN2A subunits of AMPA and NMDA receptors (Thomas and Zakharenko 2021). Consequently, miR-137’s role in glutamate signaling may be particularly relevant to the onset of schizophrenia.

The dysregulation of miR-137 and its targets supports the neurodevelopmental hypothesis of schizophrenia, suggesting that early developmental disruptions contribute to the disorder. miR-137 is highly expressed in the amygdala compared to the prefrontal cortex, implicating it in emotion processing networks. It is upregulated by metabotropic glutamate receptor 5 (mGluR5), which plays a role in emotion processing pathways. Variants near miR-137 have been linked to altered frontoamygdala connectivity, indicating its involvement in emotion dysregulation in schizophrenia (Pergola et al. 2024; Quarto et al. 2023). The dysregulation of miR-137 in emotion-processing circuits suggests its contribution to the emotional symptoms observed in schizophrenia.

The implications of miR-137 expression levels in schizophrenia are multifaceted. As a leading candidate gene for schizophrenia susceptibility identified through GWAS, variations associated with increased miR-137 expression have been linked to a higher risk of schizophrenia (Chen et al. 2021). Overexpression of miR-137 impairs synaptic transmission and plasticity, leading to cognitive deficits and psychotic symptoms. Moreover, miR-137 is crucial in neurogenesis, dendritic arborization, and neurodevelopment. Its dysregulation may result in abnormal synapse formation, contributing to schizophrenia-related cognitive impairments.

miR-137’s influence on genes involved in synaptic plasticity and neuronal development also affects executive function and emotion regulation, which are frequently impaired in schizophrenia. Given its differential expression in schizophrenia patients, miR-137 has been proposed as a potential biomarker, particularly for early-onset schizophrenia (EOS). Understanding its role in synaptic dysfunction could inform therapeutic strategies targeting schizophrenia. Additionally, evidence suggests a plausible protective effect of miR-137 overall neurodevelopment.

In summary, miR-137’s dysregulation is associated with increased schizophrenia risk, impaired synaptic function, and deficits in cognitive and emotional processing. Its expression levels may serve as a biomarker for diagnosis and a potential therapeutic target for addressing synaptic dysfunction in schizophrenia.

## miR-346 in regulating GRID1 gene in schizophrenia

miR-346 is a microRNA located within the intron of the glutamate receptor ionotropic δ1 (GRID1) gene, which has been associated with schizophrenia susceptibility. Studies have demonstrated that miR-346 expression levels are significantly lower in schizophrenia patients compared to controls. MicroRNAs, including miR-346, play a crucial role in regulating synaptic plasticity genes by modulating their expression, thereby contributing to the neurodevelopmental hypothesis of schizophrenia. This hypothesis posits that schizophrenia arises from disruptions in normal brain development, which may influence miRNA-mediated gene regulation (Wang et al. 2014; Pergola et al. 2023).

miR-346 influences synaptic plasticity in schizophrenia primarily through its association with the GRID1 gene, which is involved in schizophrenia susceptibility. Its regulatory function within glutamatergic signaling pathways suggests that dysregulation of miR-346 may lead to synaptic dysfunction, a key feature of schizophrenia.

miRNAs regulate gene expression in synaptic plasticity, essential for learning and memory. Dysregulation of these miRNAs can lead to abnormal synaptic function, contributing to schizophrenia (Wang et al. 2014).

GRID1 encodes the glutamate receptor ionotropic δ1 subunit and plays a significant role in schizophrenia susceptibility due to its involvement in glutamatergic transmission. The altered expression and genetic variations within GRID1 have been linked to schizophrenia pathology.

Schizophrenia is strongly associated with glutamatergic dysfunction, wherein genes involved in glutamate signaling, such as GRID1, are key contributors to the disorder’s etiology (Rosano et al. 2024). Several single-nucleotide polymorphisms (SNPs) within the GRID1 gene have been identified as significantly associated with schizophrenia, suggesting that GRID1 may influence the risk of developing the disorder. Variations in GRID1, particularly in its promoter region, have been linked to structural changes in the brain, such as alterations in gray matter density in the anterior thalamus and prefrontal cortex, regions implicated in schizophrenia (Nenadic et al. 2012; Ung et al. 2024).

Given that Glutamate-related excitotoxicity can affect glial cells and axons, altered glutamate metabolism is thought to contribute to the pathology of the white matter in psychotic disorders. The role of GRID1 in schizophrenia has been further supported by research showing connections between glutamatergic gene polymorphisms and white matter abnormalities, as well as elevated expression of white matter glutamate receptors in postmortem schizophrenia samples (Bryant et al. 2021).

The neurodevelopmental hypothesis posits that schizophrenia results from disruptions in brain development. miRNAs like miR-346, by regulating genes involved in synaptic plasticity and neurodevelopment, can influence the risk and progression of schizophrenia (Pergola et al. 2023). Overall, miR-346 and other miRNAs are integral components of the molecular networks that underpin the neurodevelopmental aspects of schizophrenia.

Quantitative qPCR studies have consistently demonstrated reduced miR-346 and GRID1 expression in schizophrenia patients, reinforcing the hypothesis that miR-346 plays a role in disease pathogenesis. This reduction in expression contributes to disruptions in normal glutamatergic signaling and synaptic plasticity, key processes in brain function and development (Wang et al. 2014; Dinan 2010). The lower expression of miR-346 in schizophrenia patients highlights its potential as a biomarker for the disorder.

miR-346 plays a critical role in schizophrenia pathogenesis by regulating genes involved in glutamatergic signaling and synaptic plasticity. Its dysregulation may contribute to synaptic dysfunction, neurodevelopmental disruptions, and schizophrenia progression. Furthermore, its potential as a biomarker underscores the importance of continued research into miRNA-based mechanisms in schizophrenia.

## miR-132 in neurodevelopment and synaptic plasticity

miR-132 is a microRNA that regulates neuronal differentiation, maturation, and synaptic plasticity. It is involved in axon growth, neural migration, and signaling pathways essential for neurodevelopment. In schizophrenia, miR-132 is located within genomic regions associated with the disorder and is dysregulated, contributing to neurodevelopmental and neuromorphological abnormalities. It targets genes such as DNMT3A, GATA2, and DPYSL3, which are implicated in schizophrenia pathology. This dysregulation affects synaptic plasticity and gene expression, aligning with the neurodevelopmental hypothesis of schizophrenia, suggesting that early brain development disruptions lead to cognitive and neuromorphological symptoms (Qian et al. 2017; Yoshino and Dwivedi 2020).

miR-132 exerts its effects through multiple mechanisms, particularly in regulating synaptic plasticity. It is involved in activity-dependent synaptic plasticity via NMDA receptor signaling, a critical pathway for learning and memory (Wang et al. 2022a, b). Additionally, it plays a role in neural migration and differentiation, influencing brain development and function (Ilieva 2024). Alterations in miR-132 expression in peripheral blood have also been proposed as a diagnostic biomarker for schizophrenia.

In schizophrenia, miR-132 is significantly downregulated, affecting gene expression and contributing to disease pathology. This decrease in miR-132 levels has been observed in both the prefrontal cortex of schizophrenic subjects and peripheral blood samples compared to healthy controls. The downregulation of miR-132 is associated with neurodevelopmental and neuromorphological abnormalities, affecting crucial processes such as synaptic plasticity and neuronal maturation. Overexpression of miR-132 increases the paired-pulse ratio and decreases synaptic depression in hippocampal neurons, indicating its role in modulating short-term synaptic plasticity without affecting basal release probability.

The activity-dependent synaptic formation of miR-132 is influenced by brain-derived neurotrophic factor (BDNF), which upregulates miR-132, promoting synaptic formation and plasticity. miR-132 is also involved in experience-induced synapse proteomic expression necessary for synaptic plasticity (Rashidi et al. 2023). Furthermore, its contribution to NMDA receptor function is critical for learning and memory processes. The dendritic spine stability is regulated by miR-132, as excessive levels can lead to abnormal synaptic structures. This neurite growth and arborization regulation is essential for maintaining synaptic integrity (Bludau et al. 2024). Given its role in schizophrenia, miR-132 is considered a key regulator of both short-term and long-term changes in neural connections. The administration of NMDA antagonists results in miR-132 downregulation in the prefrontal cortex; mirroring NMDA receptor hypofunction observed in schizophrenia. Additionally, miR-132 expression exhibits significant developmental regulation, coinciding with critical neurodevelopmental processes during adolescence.

### Interaction between miR-132 and miR-212 in schizophrenia

miR-132 and miR-212 are structurally related microRNAs that share a seed sequence but do not show mRNA targeting profiles that are highly overlapping. This indicates they function in a complex, non-redundant manner to shape the transcriptional profile of the central nervous system (CNS). Both miR-132 and miR-212 are implicated in synaptic plasticity and neuronal maturation, processes significantly affected in schizophrenia. Despite sharing a seed sequence, they target different mRNAs, suggesting distinct roles in CNS gene regulation. Notably, miR-132 is expressed at significantly higher levels than miR-212 in the hippocampus and cortex, implying greater functional activity in these regions. Dysregulation of both miRNAs has been linked to schizophrenia, contributing to synaptic plasticity deficits and neuronal connectivity impairments (Bormann et al. 2021; Hansen et al. 2016).

The interplay between miR-132 and miR-212 highlights their cooperative yet distinct roles in neuronal function, with their dysregulation contributing to schizophrenia’s neurodevelopmental and neuromorphological abnormalities. Epigenetic changes, such as increased expression of DNA methyltransferase alpha (DNMT3A), are associated with miR-132 dysregulation, creating a feedback loop that affects gene expression. The correlation between miR-132 and miR-212 expression levels and cognitive function in schizophrenia underscores their importance in maintaining normal synaptic plasticity and cognitive processes (Kuzniewska et al. 2022; Stojanovic et al. 2021).

Both miR-132 and miR-212 are downregulated in schizophrenia, impairing synaptic plasticity and cognitive function. This downregulation compromises neuronal connectivity and contributes to cognitive deficits. The dysregulation of these miRNAs is also linked to NMDA receptor hypofunction, a key feature of schizophrenia’s pathophysiology. Proper expression levels of miR-132 are necessary for optimal cognitive function, as both under-expression and overexpression can impair memory formation (Kouhnavardi et al. 2023; Stojanovic et al. 2022).

In conclusion, miR-132 is a key regulatory microRNA involved in synaptic plasticity, neuronal maturation, and neurodevelopment. Its dysregulation in schizophrenia is associated with synaptic dysfunction, NMDA receptor alterations, and cognitive impairments. Understanding how miR-132 and miR-212 influence neurodevelopmental processes may provide valuable insights into schizophrenia pathogenesis and potential therapeutic targets.

## miRNAs associated with cortical dysfunction in schizophrenia

### miR-181b in cortical dysregulation

The upregulation of miR-181b in cortical regions, particularly the prefrontal cortex (PFC) and superior temporal gyrus (STG), has emerged as one of the most consistent findings in miRNA research on schizophrenia. Post-mortem studies of the PFC (*n* = 21 SCZ vs. 21 healthy controls) and STG/DLPFC (*n* = 36 SCZ vs. 36 controls) demonstrate significant increases in miR-181b expression, correlating with deficits in executive function and auditory processing. Pathway analyses suggest miR-181b targets genes involved in synaptic pruning (e.g., GRIA2, NRG1) and calcium signaling, potentially disrupting glutamatergic transmission in the STG, a region critical for language perception. In the DLPFC, miR-181b overexpression may impair working memory by dysregulating dopaminergic signaling through direct inhibition of DRD1. At the same time, these findings position miR-181b as a biomarker candidate; limitations persist, including small sample sizes and a lack of longitudinal data linking miRNA changes to symptom progression (Li et al. 2024; Wang et al. 2014).

### The miR-15 family: BDNF suppression and cognitive deficits

The miR-15 family, comprising miR-15a, miR-15b, miR-16, and miR-195, exhibits pronounced dysregulation in SCZ. Peripheral blood studies of first-episode SCZ patients (n = 118) reveal elevated miR-195 levels negatively correlated with the expression of the BDNF protein. This miRNA directly binds the 3’-UTR of BDNF mRNA, repressing its translation and contributing to synaptic deficits observed in the MATRICS Consensus Cognitive Battery (MCCB) assessments (Pan et al. 2021). In the PFC, miR-16 upregulation parallels reductions in BCL2, an anti-apoptotic gene, potentiating neuronal loss through caspase-3 activation. Notably, miR-15 family members also target EGR3 and NRG1, genes implicated in SCZ risk via genome-wide association studies (GWAS), suggesting a convergent mechanism for miRNA-mediated gene silencing in cortical dysfunction (Alural et al. 2017).

### 22q11 deletion syndrome and calcium signaling

About 30% of people with 22q11 deletion syndrome (22q11DS) experience the development of SCZ, with miRNA dysregulation emerging as a key mediator. Mouse models of 22q11DS show depletion of miR-25 and miR-185, which regulate sarco/endoplasmic reticulum Ca²⁺ ATPase (SERCA2). Restoring these miRNAs in presynaptic neurons rescues long-term potentiation (LTP) deficits in the dentate gyrus, implicating calcium homeostasis in SCZ-associated cognitive impairment. Human post-mortem data further identify miR-30e and miR-346 as downregulated in the PFC of SCZ patients, with miR-346 co-expressed with GRID1, a glutamate receptor subunit linked to SCZ susceptibility. These findings underscore the interplay between copy number variations (CNVs), miRNA networks, and excitatory signaling pathways (Wang et al. 2014; Alural et al. 2017).

## miR-124 and metabolic reprogramming in early neurogenesis

miR-124 drives metabolic maturation during human neurogenesis, as demonstrated by proteomic analyses of neural progenitor cells (NPCs) differentiated from embryonic stem cells. Knockdown of miR-124 increases oxidative phosphorylation (OXPHOS) proteins (e.g., GSTK1, NDUFS1) while reducing mitochondrial membrane potential (ΔΨm) by 40% (*p* < 0.01). These metabolic shifts correlate with stunted neurite outgrowth (mean length reduction: 28%, *p* < 0.05) and diminished electrophysiological activity in cortical neurons. Notably, 11 OXPHOS proteins altered by miR-124 depletion overlap with pathways implicated in Alzheimer’s and Parkinson’s diseases, suggesting shared mechanisms of metabolic dysfunction (Son et al. 2024).

miR-124 depletion exacerbates mitochondrial dysfunction, reducing ATP production by 35% (*p* < 0.01) in differentiating neurons. Concurrent increases in reactive oxygen species (ROS) activate JNK/p38 pathways, promoting neuronal apoptosis (Son et al. 2024). In SCZ, oxidative stress markers (e.g., 8-OHdG) correlate with miR-124 levels in cerebrospinal fluid (*r* = 0.68, *p* < 0.01), suggesting a feedback loop between metabolic impairment and neurodegeneration (Alural et al. 2017; Son et al. 2024).

## miR-15/16-mediated regulation of BCL2 and apoptosis

The miR-15 family’s targeting of *BCL2* provides a mechanistic link between miRNA dysregulation and cerebral atrophy in SCZ. Post-mortem PFC samples show miR-16 upregulation coinciding with 50% reductions in BCL2 protein (*p* < 0.01) (Li et al. 2024; Alural et al. 2017). This pro-apoptotic shift increases caspase-3 activity by 3.2-fold (*p* < 0.001), mirroring findings in peripheral blood mononuclear cells (PBMCs) of SCZ patients (Pan et al. 2021). Longitudinal MRI studies associate miR-15/16 overexpression with accelerated gray matter loss in the STG (*r* = −0.71, *p* < 0.05), supporting their role in disease progression (Li et al. 2024).

This evidence implicates microRNAs (miRNAs) as critical regulators of these processes, with specific miRNAs showing dysregulation in brain regions central to SCZ pathology, including the prefrontal cortex (PFC), superior temporal gyrus (STG), and dorsolateral prefrontal cortex (DLPFC). This synthesizes recent findings on miR-181b, miR-16, miR-20a, miR-124, and the miR-15 family, highlighting their roles in cortical dysfunction, neurogenesis, apoptosis, and synaptic signaling. Key studies reveal upregulated miR-181b in the PFC and STG of SCZ patients, altered miR-15/195 expression impacting brain-derived neurotrophic factor (BDNF) signaling, and miR-124-driven metabolic reprogramming during neurogenesis. These miRNAs collectively contribute to the progressive brain tissue loss and cognitive deficits observed in SCZ, offering a novel diagnostic avenue.

**Fig. 4:**
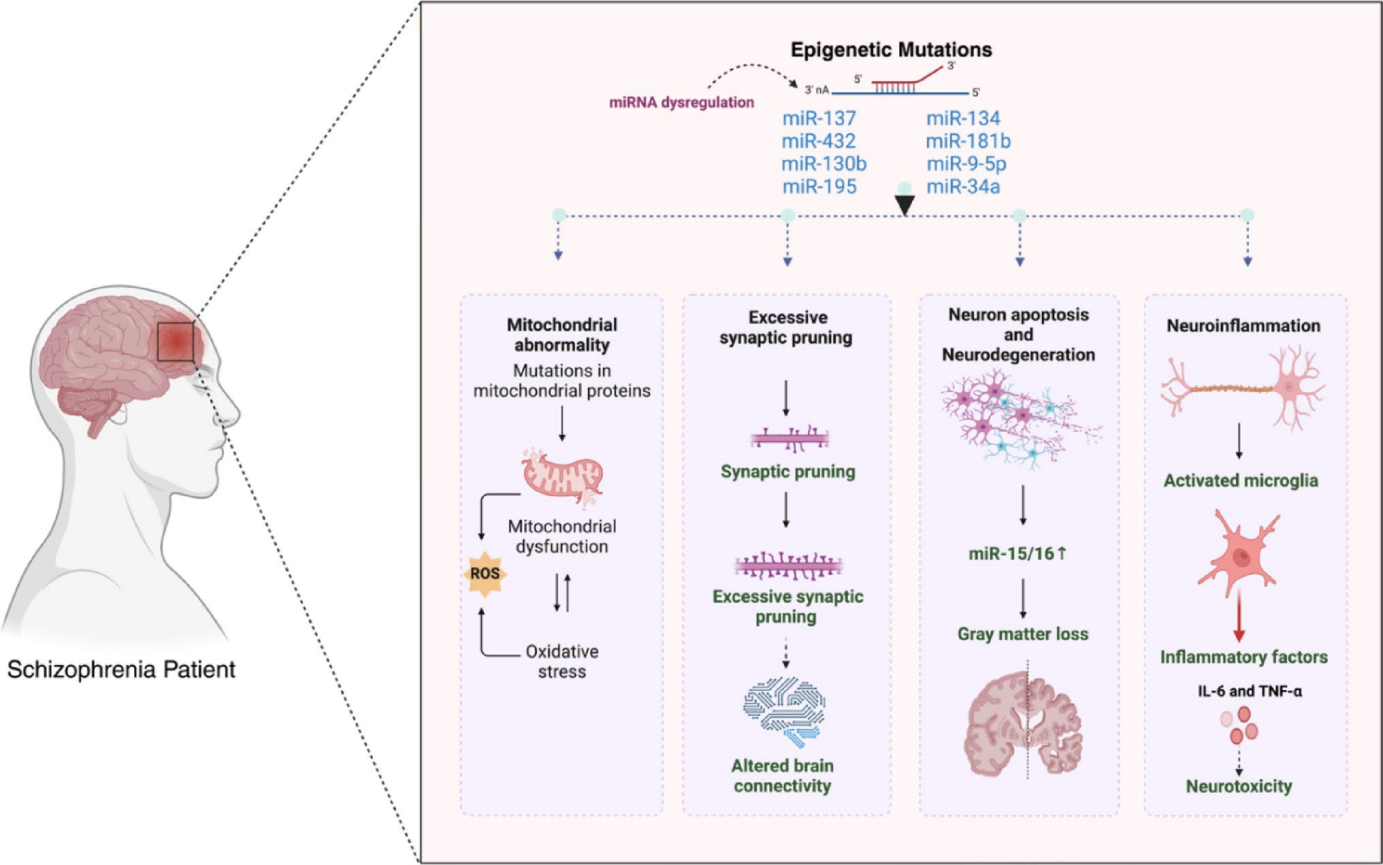
The impact of epigenetic mutations and miRNA dysregulation on schizophrenia pathophysiology. Schematic representation of neuronal dysfunction in schizophrenia highlights epigenetic dysregulation’s effects (e.g., miR-137, miR-34a, and others) on brain pathology. This dysregulation results in multiple pathological features observed in schizophrenia, including neurodegeneration (progressive neuronal loss and reduced brain volume), neuroinflammation (overactive microglia releasing pro-inflammatory cytokines), and excessive synaptic pruning (loss of functional synapses). Additionally, mitochondrial dysfunction and oxidative stress contribute to neuronal energy deficits, further exacerbating cognitive and structural impairments in schizophrenia. These interconnected mechanisms collectively drive synaptic and neuronal dysfunction, leading to the characteristic symptoms of the disorder

Recent research has increasingly focused on the role of microRNAs (miRNAs) in schizophrenia, highlighting their potential as diagnostic biomarkers and therapeutic targets. This table summarizes key case-control and meta-analytic studies that have examined the differential expression of miRNAs in schizophrenia patients compared to healthy controls Table 2. The studies included span diverse populations and utilize various methodological approaches, such as qRT-PCR, TLDA, and miRNA arrays, to validate miRNA expression levels. Many of these miRNAs, including miR-34a, miR-132, miR-181b, and miR-137, have been linked to neurodevelopmental processes, synaptic plasticity, and apoptotic pathways—key mechanisms implicated in schizophrenia pathology (Fig. 4). Additionally, some studies explore clinical correlations between miRNA expression and symptom severity, neurocognitive performance, and treatment response. Understanding these dysregulated miRNAs and their functional implications could pave the way for more objective diagnostic tools and novel therapeutic strategies in schizophrenia.

**Table 2:** Summary of Case-Control and Meta-Analytic studies investigating MiRNA dysregulation in schizophrenia

Study design	Population Demographics	miRNAs Studied	Clinical Correlation/Diagnosis	Testing Method	Findings	Citation
A case-control study with validation in an independent testing set	90 schizophrenia patients, 60 healthy controls	hsa-miR-34a, hsa-miR-449a, hsa-miR-564, hsa-miR-432, hsa-miR-548d, hsa-miR-572, hsa-miR-652	Correlated with negative symptoms, neurocognitive performance scores, and event-related potentials	TaqMan Low-Density Array (TLDA) and quantitative reverse transcription polymerase chain reaction (qRT-PCR)	Identified a seven-miRNA signature for schizophrenia with 85% AUC in validation. • miR-34a was significantly differentially expressed between cases and controls in both the learning set (*P* = 0.005) and testing set (*P* = 0.002). Predicted target genes of these miRNAs were significantly associated with nervous system development and function, including the Cdk5, Notch signaling, and dopamine receptor signaling pathways.	(Lai et al. 2011)
Meta-analysis of case-control studies and a validation case-control study	330 schizophrenia patients, 202 healthy controls. 39 schizophrenia patients, 50 healthy controls; Chinese Han nationality	miR-181b-5p, miR-21-5p, miR-195-5p, miR-137, miR-346, miR-34a-5p	All extracted data were statistically analyzed, and the results were further validated with peripheral blood mononuclear cells (PBMNCs) isolated from patients and healthy controls.	qRT-PCR	The pooled sensitivity and specificity of miRNAs as diagnostic markers for schizophrenia were both 0.81, indicating good diagnostic performance. The area under the summary receiver operating characteristic (ROC) curve was 0.87, demonstrating high diagnostic accuracy. The positive likelihood ratio was 4.3 and the negative likelihood ratio was 0.24, supporting the test’s clinical utility. The combined diagnostic odds ratio (DOR) was 18, highlighting the strong discriminatory ability of miRNAs in schizophrenia diagnosis. Validation confirmed that six specific miRNAs exhibited high diagnostic sensitivity and specificity. Leave-one-out support vector machine analysis of these six miRNAs achieved a diagnostic accuracy of 86.37%, with a true positive rate of 81.76%, true negative rate of 93.43%, false positive rate of 8.24%, and false negative rate of 6.57%.	(Liu et al. 2017a, b)
Case-control study	57 schizophrenia patients, 62 healthy controls	miR-320d	Relationship with MCCB score in both control and schizophrenia groups	qRT-PCR	Downregulated serum miR-320d was identified in schizophrenia and remained independently associated with the disorder after adjusting for confounders. Serum miR-320d correlated with MCCB cognitive scores in both patients and controls and distinguished schizophrenia cases from controls with an AUC of 0.931. Predicted target genes of miR-320d were enriched in cell adhesion, GTPase activity, and the Rap1 signaling pathway.	(Ren et al. 2024)
Analytical, case-controlled, cross-sectional study	650 schizophrenia patients, 924 healthy controls	hsa-miR-34a, miR-449a, miR-564, miR-432, miR-548d, miR-572, miR-652	Relation with gender differences, the impact of smoking and alcohol consumption, family history of schizophrenia, refractoriness of the Disorder, and the Subtypes of schizophrenia.	qRT-PCR	All seven miRNAs were significantly different between schizophrenia patients and controls (*p* < 0.05). miR-449a was significantly associated with the paranoid subtype compared to other subtypes (*p* < 0.05).	(Rodrigues et al. 2024)
Case-control study	30 schizophrenia patients, 35 healthy controls; Jordanian cohort	miR-29b-3p, miR-106b-5p, miR-199a-3p	key biological processes affected by miRNAs: neuronal development, morphogenesis, Synaptic Plasticity, Regulation of Apoptosis, and Cellular Signaling Pathways	qRT-PCR	*miR-106b-5p* and *miR-199a-3p* were significantly upregulated in schizophrenia patients (*p* < 0.0001 for both). *miR-29b-3p* was significantly downregulated in schizophrenia patients (*p* < 0.0001). *miR-199a-3p* showed high diagnostic value with an AUC of 0.979. *miR-106b-5p* showed diagnostic value with an AUC of 0.774. *miR-29b-3p* exhibited limited diagnostic efficacy.	(Shboul et al. 2024)
Cross-sectional observational study with a prospective cohort component	61 schizophrenia patients, 62 healthy controls	miR-30e, miR‐181b, miR‐34a, miR‐346, miR‐7, miR‐132, miR‐432, miR‐212	Correlated with clinical symptomatology improvements and treatment responses	qPCR	A panel of miR-30e, miR-181b, miR-34a, miR-346, and miR-7 was significantly upregulated in schizophrenia patients and showed diagnostic value (AUC: 0.713, sensitivity: 35.5%, specificity: 90.2%). After six weeks of antipsychotic treatment, expression levels of *miR-132*, *miR-181b*, *miR-432*, and *miR-30e* significantly decreased. Reductions in *miR-132*, *miR-181b*, *miR-212*, and *miR-30e* expression were significantly correlated with improvements in clinical symptoms. Decreases in plasma *miR-132* and *miR-432* were significantly greater in patients with high treatment response compared to low-effect subgroup after six weeks.	(Sun et al. 2015)
A multistage case-control study with prospective cohort follow-up	564 schizophrenia patients, 400 healthy controls	miR-130b, miR-193a-3p	Correlated with the control group.	Solexa sequencing, Taqman Low-Density Array (TLDA), (qRT-PCR)	Eight miRNAs were significantly upregulated in schizophrenia patients. Among them, *miR-130b* and *miR-193a-3p* were identified by qRT-PCR as state-independent biomarkers for schizophrenia.	(Wei et al. 2015)
Case-control study	60 schizophrenia patients, 72 healthy controls	miR-30e, miR-34a, miR-181b, miR-195, miR-346, miR-432, miR-7, miR-132, miR-212	Investigated the differences in the expression levels of microRNA (miRNA or miR) in plasma and peripheral blood mononuclear cells of patients with schizophrenia.	Real-Time PCR	*miR-132*, *miR-195*, *miR-30e*, and *miR-7* showed significantly higher expression in plasma of schizophrenia patients compared to controls (*P* < 0.05, *P* < 0.001) *miR-212*, *miR-34a*, and *miR-30e* also showed significantly higher expression in mononuclear leukocytes of patients compared to controls (*P* < 0.05, *P* < 0.001). Plasma *miR-30e*: AUC 0.767 (95% CI: 0.608–0.926), sensitivity 90.9%, specificity 60%. Mononuclear leukocyte *miR-30e*: AUC 0.756 (95% CI: 0.584–0.929), sensitivity 81.8%, specificity 68%.	(Zhang et al. 2014)
Secondary data analysis, meta-analysis, and case-control study	40 schizophrenia patients, 40 healthy controls.	hsa-miR-574 5P, hsa-miR-1827, hsa-miR-4429	Correlated in the blood samples of schizophrenia patients compared to healthy controls.	miRNA array and Real-Time PCR	*hsa-miR-574-5p*, *hsa-miR-1827*, and *hsa-miR-4429* were significantly dysregulated in the blood of schizophrenia patients and proposed as potential diagnostic biomarkers. Several genes (*CREBRF*, *ARPP19*, *TGFBR2*, *YWHAZ*) were also found to be dysregulated in schizophrenia blood samples.	(Davarinejad et al. 2022)
Case-control study	38 schizophrenia patients, 50 healthy controls	miR-30a-5p, miR-30c-5p, miR-30e-5p	The EGR1, miR-30a-5p, and NEUROD1 axis a potential biomarkers for schizophrenia diagnosis and treatment monitoring.	Quantitative Polymerase Chain Reaction (qPCR)	In PBMNCs from schizophrenia patients, EGR1 and miR-30a-5p were significantly downregulated, while NEUROD1 was significantly upregulated, compared to controls. After 12 weeks of antipsychotic treatment, EGR1 and miR-30a-5p levels increased, and NEUROD1 levels decreased. The increase in *miR-30a-5p* was significantly correlated with a reduction in negative symptoms (*r* = − 0.363, *p* < 0.05). The EGR1–miR-30a-5p–NEUROD1 axis had high diagnostic value (AUC = 0.962), outperforming *miR-30a-5p* alone (AUC = 0.649); sensitivity: 83.3%, specificity: 97.9%. All 30 treated patients showed significant improvements in total, positive, negative, and general psychopathology PANSS scores (*p* < 0.001).	(Liu et al. 2017a, b)
Case-control study	40 schizophrenia patients, 40 healthy controls	miR-34a-5p, miR-432-5p, miR-449a	Correlated in the serum samples of schizophrenia patients compared to healthy controls.	qRT-PCR	*miR-34a-5p*, *miR-432-5p*, and *miR-449a* were significantly dysregulated in the serum of schizophrenia patients compared to controls. Among individual miRNAs, miR-432-5p showed the best diagnostic performance: AUC: 0.764, sensitivity: 62.5%, specificity: 82.5%. Combining miRNAs improved diagnostic accuracy: *miR-34a-5p + miR-432-5p*: AUC: 0.807, sensitivity: 97.5%, specificity: 55%, Youden index: 0.525. *miR-432-5p + miR-449a*: AUC: 0.841, sensitivity: 90%, specificity: 80%, Youden index: 0.7. *miR-34a-5p + miR-432-5p + miR-449a*: AUC: 0.843, sensitivity: 90%, specificity: 77.5%, Youden index: 0.675.	(He et al. 2019)

## Challenges and perspectives

Despite the growing evidence supporting the role of miRNAs in schizophrenia, several challenges hinder their clinical translation. One major limitation is the variability in study methodologies, including differences in sample types (plasma, serum, cerebrospinal fluid, or brain tissue), patient populations, and miRNA profiling techniques. These inconsistencies complicate the reproducibility and comparability of findings across studies. Additionally, the specificity of miRNAs as biomarkers remains a concern, as many miRNAs implicated in schizophrenia also overlap with other neuropsychiatric disorders such as bipolar disorder and major depressive disorder. Another challenge is the dynamic regulation of miRNA expression, which can be influenced by medication, disease progression, and environmental factors, making it difficult to establish standardized diagnostic thresholds.

Future research should focus on large-scale, longitudinal studies to validate miRNA-based biomarkers and their clinical utility. Advancements in single-cell sequencing and bioinformatics approaches may help identify more specific miRNA signatures associated with schizophrenia subtypes and disease progression. Furthermore, integrating miRNA profiling with other omics data, such as transcriptomics and proteomics, could provide a more comprehensive understanding of schizophrenia pathology. Developing targeted miRNA-based therapies, such as miRNA mimics or inhibitors, also holds promise for personalized treatment strategies, potentially improving patient outcomes and addressing cognitive deficits more effectively.

## Conclusion

The dysregulation of miRNAs in schizophrenia represents a promising avenue for understanding the molecular underpinnings of the disorder and developing objective diagnostic tools. Numerous studies have identified key miRNAs associated with neurodevelopment, synaptic plasticity, and apoptosis, linking their dysregulation to cognitive dysfunction and disease progression. However, methodological variability, specificity concerns, and external influences on miRNA expression must be addressed before miRNAs can be reliably used in clinical practice. Future research efforts should focus on refining biomarker panels, validating findings across diverse populations, and exploring therapeutic interventions targeting miRNA dysregulation. By overcoming these challenges, miRNA-based diagnostics and therapeutics may revolutionize schizophrenia management, leading to earlier detection and more effective treatments.

## Data Availability

All data generated are present in the current MS.

## Abbreviations

A-to-I: Adenosine-to-Inosine
ADAR: Adenosine Deaminase Acting on RNA
APOBEC: Apolipoprotein B mRNA Editing Enzyme Catalytic Subunit
BDNF: Brain-Derived Neurotrophic Factor
CACNA1C: Calcium Voltage-Gated Channel Subunit Alpha1 C
C-to-U: Cytosine-to-Uridine
circRNA: Circular RNA
CNVs: Copy Number Variations
COMT: Catechol-O-Methyltransferase
DLPFC: Dorsolateral Prefrontal Cortex
DNMT3A: DNA Methyltransferase 3 Alpha
DGCR8: DiGeorge Syndrome Critical Region 8
DRD2: Dopamine D2 Receptor
DSM-5: Diagnostic and Statistical Manual of Mental Disorders, Fifth Edition
EGR1: Early Growth Response 1
FXR1: Fragile X Mental Retardation Protein 1
GABA: Gamma-Aminobutyric Acid
GRID1: Glutamate Receptor Ionotropic Delta 1
GSK3B: Glycogen Synthase Kinase 3 Beta
ICD-11: International Classification of Diseases, 11th Revision
JNK: c-Jun N-Terminal Kinase
lncRNA: Long Non-Coding RNA
LTP: Long-Term Potentiation
LTD: Long-Term Depression
MATRICS: Measurement and Treatment Research to Improve Cognition in Schizophrenia
MCCB: MATRICS Consensus Cognitive Battery
mGluR: Metabotropic Glutamate Receptor
miRNA: MicroRNA
mRNA: Messenger RNA
MCCB: MATRICS Consensus Cognitive Battery
NRGN: Neurogranin
NMDA: N-Methyl-D-Aspartate
OXPHOS: Oxidative Phosphorylation
PANSS: Positive and Negative Syndrome Scale
PBMCs: Peripheral Blood Mononuclear Cells
PBMNCs: Peripheral Blood Mononuclear Cells
PFC: Prefrontal Cortex
PSD-95: Postsynaptic Density Protein 95
qPCR: Quantitative Polymerase Chain Reaction
qRT-PCR: Quantitative Reverse Transcription Polymerase Chain Reaction
RISC: RNA-Induced Silencing Complex
ROS: Reactive Oxygen Species
SCZ: Schizophrenia
SERCA2: Sarco/Endoplasmic Reticulum Calcium ATPase 2
SHANK: SH3 and Multiple Ankyrin Repeat Domains Protein
SNP: Single Nucleotide Polymorphism
STG: Superior Temporal Gyrus
TLDAs: Taqman Low Density Arrays
TMT-B: Trail Making Test-Part B; WAIS-III: Wechsler Adult Intelligence Scale-Third Edition
WCST: Wisconsin Card Sorting Test
ΔΨm: Mitochondrial Membrane Potential
22q11DS: 22q11 Deletion Syndrome
